# In-Depth Quantitative Proteomics Characterization of In Vitro Selected Miltefosine Resistance in *Leishmania infantum*

**DOI:** 10.3390/proteomes10020010

**Published:** 2022-03-31

**Authors:** Leonardo Saboia-Vahia, Patricia Cuervo, Jacek R. Wiśniewski, Geovane Dias-Lopes, Nathalia Pinho, Gabriel Padrón, Fernando de Pilla Varotti, Silvane Maria Fonseca Murta

**Affiliations:** 1 Grupo de Genômica Funcional de Parasitos (GFP), Instituto René Rachou, Fiocruz Minas, Belo Horizonte 30190-002, MG, Brazil; 2 Laboratório de Pesquisa em Leishmanioses, Instituto Oswaldo Cruz, Fiocruz, Rio de Janeiro 21040-360, RJ, Brazil; patricia.cuervo@fiocruz.br (P.C.); nathps@ioc.fiocruz.br (N.P.); gpadronpalomares@gmail.com (G.P.); 3 Rio de Janeiro Research Network on Neuroinflammation, Oswaldo Cruz Institute, Oswaldo Cruz Foundation, Rio de Janeiro 21040-360, RJ, Brazil; 4 Biochemical Proteomics Group, Department of Proteomics and Signal Transduction, Max-Planck-Institute of Biochemistry, 82152 Planegg, Germany; jwisniew@biochem.mpg.de; 5 Laboratório de Biologia Molecular e Doenças Endêmicas, Instituto Oswaldo Cruz, Fiocruz, Rio de Janeiro 21040-360, RJ, Brazil; geovane.dl@gmail.com; 6 Research Center on Biological Chemistry (NQBio), Federal University of São João Del Rei, Divinópolis 35501-296, MG, Brazil; varotti@ufsj.edu.br

**Keywords:** *Leishmania infantum*, miltefosine resistance, quantitative proteomics, FASP, mass spectrometry, oxidative phosphorylation, fatty acid β-oxidation, cytochrome *c* oxidase, ATP synthase

## Abstract

Visceral leishmaniasis (VL) is a neglected disease caused by *Leishmania* parasites. Although significant morbidity and mortality in tropical and subtropical regions of the world are associated with VL, the low investment for developing new treatment measures is chronic. Moreover, resistance and treatment failure are increasing for the main medications, but the emergence of resistance phenotypes is poorly understood at the protein level. Here, we analyzed the development of resistance to miltefosine upon experimental selection in a *L. infantum* strain. Time to miltefosine resistance emergence was ~six months and label-free quantitative mass-spectrometry-based proteomics analyses revealed that this process involves a remodeling of components of the membrane and mitochondrion, with significant increase in oxidative phosphorylation complexes, particularly on complex IV and ATP synthase, accompanied by increased energy metabolism mainly dependent on β-oxidation of fatty acids. Proteins canonically involved in ROS detoxification did not contribute to the resistant process whereas sterol biosynthesis enzymes could have a role in this development. Furthermore, changes in the abundance of proteins known to be involved in miltefosine resistance such as ABC transporters and phospholipid transport ATPase were detected. Together, our data show a more complete picture of the elements that make up the miltefosine resistance phenotype in *L. infantum*.

## 1. Introduction

Visceral leishmaniasis (VL) is a neglected disease that frequently afflicts the most vulnerable populations living in tropical and subtropical regions of the world. The disease is caused by protozoan parasites of the *Leishmania* genus, mainly *Leishmania donovani* in Asia and Africa and *L. infantum* in the Mediterranean Basin, central Asia, Middle East and Latin America [1,2]. In both the Mediterranean Basin and Latin America, dogs are the main host, playing an important role in parasite’ transmission cycle. Although the incidence of VL has reduced considerably over the last decade, 90,000 new cases were reported in 2017 with more than 90% of the cases occurring in 10 countries: Brazil, China, Ethiopia, Eritrea, India, Kenya, Somalia, South Sudan, Sudan and Yemen [1,3,4]. In Latin America, Brazil reports more than 97% of the cases in the region per year [5]. The systemic disease caused by those parasites is characterized by persistent fever, splenomegaly, weight loss and anemia, and is fatal if not properly treated. It mainly affects children under five years of age, undernourished people and individuals with other conditions of immunosuppression [6,7]. 

Depending on the clinical conditions of the patients, treatment of VL is made with a reduced chart of medications limited to pentavalent antimonials, paromomycin, miltefosine and amphotericin B (in free deoxycholate form and lipid formulations). For more than six decades, pentavalent antimonial monotherapy has been the first-line treatment for VL, but, due to the emergence of resistance to this medication, its use is no longer recommended in various regions of the world [8,9]. New treatments include injectable paromomycin, a single dose of liposomal amphotericin B and oral miltefosine or combined regimes of those medicines [7,9]. 

Miltefosine (Hexadecylphosphocholine) is an alkyl phospholipid originally developed for the treatment of cutaneous metastasis of breast carcinomas and is currently the only oral medication for the treatment of human VL [9]. As of 2002, the drug was approved and registered for the treatment of human VL in India, with a cure rate of 98% [10]. Furthermore, in 2014, miltefosine was approved by the US FDA as the first oral medicine for the treatment of cutaneous and mucocutaneous leishmaniasis caused by New World *Leishmania* species based, among others, on studies of efficacy conducted in Brazil against *L. braziliensis* and *L. guyanensis* [11,12,13]. Although its mechanism of action is not fully understood, it has been observed that miltefosine is incorporated into the lipid bilayer of the parasites’ plasma membrane changing its permeability, causes alterations in lipid metabolism and induces cell death by an apoptosis-like process [14,15]. Since 2007, miltefosine was registered for the oral treatment of canine VL in several European countries, and the same formulation was recently authorized by the Brazilian government for the treatment of dogs with VL [16]. This approval causes concern given that, despite the improvement in clinical symptoms, treated dogs continue with detectable levels of parasites in the blood and skin, and therefore could continue to be a source of transmission of *L. infantum* [17]. In addition, there is a real possibility of resistance emergence that might result in cross-resistance to pentavalent antimoniate and amphotericin B, which are still the first-line treatment options for human VL in Brazil [18,19]. 

After a decade of adoption of miltefosine as the first-line regimen for human VL in Asia, reduced effectiveness was reported as well as increased tolerance and treatment failure [20,21,22,23]. In Brazil, although there are few studies on this drug, the observed cure rate of human VL was between 43% to 67% in 28 day or 42 day long regimes, respectively, suggesting intrinsic resistance of the *L. infantum* to miltefosine [24]. Comparison of genome sequences of pretreatment parasites isolated from VL patients either cured or relapsing after miltefosine treatment revealed the absence of a region on chromosome 31 encoding four genes (two 3′-nucleotidase/putative nucleases, one helicase-like and a 3,2-trans-enoyl-CoA isomerase) in the isolates from patients with relapse, whereas it was present in the isolates from cured patients [25]. In further studies, it was suggested that this genomic region, named miltefosine sensitivity locus (MSL), confers natural resistance to the drug [26]. Although many Brazilian *L. infantum* isolates do not carry the MSL [27], its association with miltefosine resistance in vitro is not clear [28]. 

The mechanisms underlying miltefosine resistance are not yet well understood. Genomic characterization of Old World *L. infantum* strains with either natural or selected miltefosine resistance revealed mutations in the MT and Ros3 genes, which encode proteins involved in the transport of the drug. Such mutations result in the inactivation of the MT/Ros3 transporter complex and are considered as the main mechanism for miltefosine resistance both in promastigotes and amastigotes [29]. However, such mutations were not observed in natural miltefosine-resistant *L. donovani* isolates from VL relapse cases [30]. Interestingly, miltefosine-resistant *L. infantum* parasites exhibit important fitness loss mediated by incomplete metacyclogenesis, decreased intracellular proliferation and diminished stress response, all of which are rescued by the treatment with the drug [31,32]. Those observations suggest that mechanisms triggering miltefosine resistance are much more complex. 

Proteomics studies of drug resistance in *Leishmania* spp. have identified a set of proteins potentially involved in this phenotype and suggest the existence of a common group of proteins involved in the mechanisms of resistance to both miltefosine and antimonial [24,33,34,35,36,37,38,39,40]. However, an in-depth quantitative proteomics study of miltefosine resistance is lacking. To understand the mechanisms involved in the emergence of resistance, here we experimentally selected resistance to miltefosine in a wild-type *L. infantum* strain and conducted an unbiased and comprehensive quantitative proteomics analysis of drug-resistant parasites. Our dataset provides the first comprehensive quantitative analysis of the miltefosine resistance phenotype in terms of protein concentration and copy numbers and demonstrates the main differences in protein abundance between wild-type and resistant parasites. Data are available via ProteomeXchange with identifier PXD031912.

## 2. Materials and Methods

### 2.1. Parasite Culture, Growth Curve and Susceptibility Test

We used the *L. infantum* reference strain MHOM/BR/1974/PP75. Promastigotes were maintained at 26 °C in Schneider’s medium (Sigma-Aldrich, St. Louis, MO, USA) supplemented with 10% heat-inactivated fetal bovine serum (FBS) (Sigma-Aldrich, St. Louis, MO, USA). For analyzing the growth curve, promastigotes (1 × 10^5^ parasites) were inoculated in Schneider’s medium supplemented as above, and parasite density was determined every 24 h for two weeks using hemocytometer and light microscopy. Promastigotes of logarithmic phase were used for all experiments. 

To evaluate miltefosine cytotoxicity, 2 × 10^6^ parasites in culture medium were added to a 96-well tissue culture plate and grown in the presence of medium containing different miltefosine concentrations (ranging from 240 µM to 1.2 µM, Sigma-Aldrich) or medium without drug (control). Plates were incubated for 64 h at 26 °C and then resazurin reagent (Invitrogen, Carlsbad, CA, USA) was added and plates were incubated for a further 8 h at 26 °C. The optical density of the samples was measured in a spectrophotometer (SpectraMax—Molecular Devices, San Jose, CA, USA) at wavelengths of 540 nm and 630 nm. The effect of drug treatment on cell viability was expressed as IC_50_/72 h, which corresponds to a concentration that led to 50% lysis of the parasites within 72 h. Three independent experiments were performed in triplicate. 

### 2.2. Selection of Resistance 

For the in vitro selection of resistance, promastigotes were cultivated with increasing concentrations of miltefosine (1.2, 2.4, 4, 8, 16 and 25 µM) added in a stepwise manner, according to protocols previously described [41], totalizing approximately 6 months and 25 passages. The wild-type (WT) parasites were maintained during the same passage numbers as the resistant line but without drug pressure. The resistant line (LiR) was maintained under the continuous presence of the drug. The growth profile and IC_50_ of LiR line were determined as described above.

### 2.3. Sample Preparation and LC-MS/MS Analysis

For proteomic analysis, WT and LiR promastigotes were grown in culture medium without the drug, collected at logarithmic stage, washed three times with PBS and resuspended in lysis buffer. Four independent biological replicates of *L. infantum* resistant line (LiR) and three independent biological replicates of *L. infantum* parent wild-type strain (WT) were analyzed. Proteomics sample preparation followed protocols previously described [42,43]. Briefly, parasites were lysed in a buffer containing 0.05 M Tris-HCl (pH 7.6) (Bio-Rad, Hercules, CA, USA), 0.05 M DTT (Bio-Rad, Hercules, CA, USA) and 2% SDS (*w*/*v*) (Bio-Rad, Hercules, CA, USA) and boiled in a water bath for 5 min. After chilling to room temperature, the SDS lysates were clarified by centrifugation at 10,000× *g* for 5 min and total protein concentration was determined by BCA using a Nanodrop^®^ instrument (Thermo-Fisher Scientific, Palo Alto, CA, USA ). Protein samples (100 µg total protein) were processed in 30k filtration units (Millipore, Burlington, MA, USA) by the multi-enzyme digestion—filter aided sample preparation (MED-FASP) method using consecutively endoproteinase LysC and trypsin in a 1/100 enzyme to protein ratio [44,45]. Peptides were collected, concentrated and desalted on a C18 reversed phase column. 

For LC/MS/MS analysis, 2 μg peptide mixtures were fractionated on a reversed phase column (50 cm × 75 μm inner diameter) packed with 1.8 μm diameter C18 particles (100 Å pore size; Dr. Maisch, Ammerbuch-Entringen, Germany) using a 105 min acetonitrile (Tedia^®^, Fairfield, OH, USA) gradient in 0.1% formic acid (Sigma-Aldrich, St. Louis, MO, USA) at a flow rate of 250 nL/min. The backpressure varied between 450 and 650 bar. Peptide masses were analyzed using a Q-Exactive HF mass spectrometer (Thermo-Fisher Scientific, Palo Alto, CA, USA) operated in data-dependent mode with survey scans acquired at a resolution of 50 000 at *m*/*z* 400 (transient time 256 ms). The top 12 most abundant isotope patterns with charge ≥+2 from the survey scan (300–1650 *m*/*z*) were selected with an isolation window of 1.6 *m*/*z* and fragmented by HCD with normalized collision energies of 25. The maximum ion injection time for the survey scan was 20 ms and for the MS/MS scans was 60 ms. The ion target values for MS1 and MS2 scan modes were set to 3 × 10^6^ and 1 × 10^5^, respectively. The dynamic exclusion was 25 s and 10 ppm.

### 2.4. Data Analysis

Data were analyzed using the Andromeda search engine included in the MaxQuant Software (Ver. 1.2.6.20). The mass spectra were searched against a database containing *L. infantum* sequences available at UniProtKB/Swiss-Prot (downloaded in November 2021) plus reversed proteins used as decoys. The option “matching between runs” was used for searching; the fragment ion mass tolerance was set at 0.5 Da and parent ion tolerance at 20 ppm. The maximum false peptide and protein discovery rate was set as 1%. Cysteine carbamidomethylation was set as fixed modification, methionine oxidation as variable modification, and up to two missed cleavages were allowed. Protein absolute abundances were calculated based on the spectral protein intensity (raw intensities) using the ‘total protein approach’ (TPA), and absolute protein copy numbers per cell were estimated using the ‘proteomic ruler’ approach [46]. Calculations of total protein, protein concentration and copy number were performed in Microsoft Excel. Perseus software (Ver. 1.6.5.0) [47] was used for data validation and statistical analysis of differences in protein abundances. The minimal number of valid values was set to 3 per protein in at least one group, and missing values were imputed from a normal distribution. Significances were calculated using the *t*-test with a threshold of false discovery rate (FDR) of 3%. Data are presented as the mean ± SD. Mass spectrometry proteomics data were deposited to the ProteomeXchange Consortium via the PRIDE [48] partner repository with the dataset identifier PXD031912.

### 2.5. Enrichment Analysis Based on Gene Ontology and Metabolic Pathway Annotations

In order to analyze if there were particular groups of proteins that contributed the most for the resistant phenotype selected in vitro, only the proteins that presented significant differences in concentration between the WT and LiR parasites were used for enrichment analysis of gene ontology and metabolic pathway annotations using the enrichment tool at Tritrypdb (http://tritrypdb.org, v55, accessed on 5 February 2022). Parameters were set as follows: organism, *L. infantum* JPCM5, evidence computed and curated and *p*-value cutoff, 0.05. The search was performed for the ontology of Biological Process, Cellular Component and Molecular Function. In addition, differential proteins were mapped for *L. infantum* JPCM5 (T number: T01112) in the Kyoto Encyclopedia of Genes and Genomes (KEGG) PATHWAY database [49]. 

### 2.6. Statistical Analysis

Analyses were performed with GraphPad Prism version 8.0 for Windows (GraphPad Software, San Diego, CA, USA). Asterisks indicate significant differences with the threshold for significance set at *p* ≤ 0.05. Student’s *t* test was used to analyze the statistical significance between the WT strain and LiR line. 

## 3. Results and Discussion

### 3.1. In Vitro-Selected Miltefosine-Resistant L. infantum Line LiR Exhibits Reduced Drug Sensitivity and Has a Lower Rate of Growth Than the WT Strain

**Figure 1:**
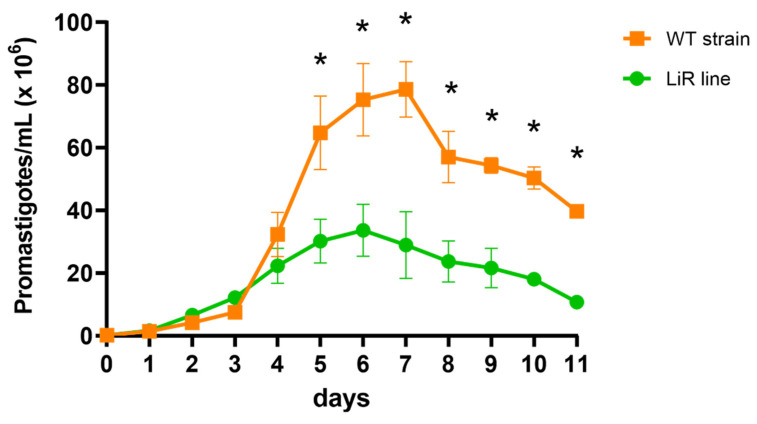
In vitro promastigote growth curves of *L. infantum* wild-type (WT) line and miltefosine-resistant (LiR) line. LiR parasites show decreased growth compared with WT line. Results are expressed as mean ± SD of three independent experiments each one made in triplicate. Significant differences between parasite WT and LiR lines, by day, were determined by two-way ANOVA followed by Sidak’s multiple comparisons test (* *p* < 0.0001).

Experimental resistance to miltefosine was selected by consecutive passages of promastigotes under drug pressure. It takes approximately six months and 25 passages to reach the highest 50% inhibitory concentration, IC_50_, 25.27 ± 1.40 µM, and resistance was stable in the absence of the drug after five passages as well as after cryopreservation. In comparison, the IC_50_ of miltefosine for the parental *L. infantum* strain was 7.4 ± 1.56 µM and this value was not altered in the WT line maintained without drug pressure for the same time of culture than the LiR line. Thus, the IC_50_ increase corresponded to a resistance index of 3.4. In addition, we looked at the in vitro growth profile of the WT and LiR promastigotes. Although the growth curves of both WT strain and LiR line exhibit logarithmic and stationary phases, the LiR line showed significant lower growth rate than the WT strain (Figure 1). These results agree with the decrease in the growth rate observed in promastigotes of *L. infantum* strains selected for miltefosine resistance as well as in other *Leishmania* species selected for resistance to different drugs [31,32,50]. This phenotype has been associated with a decreased fitness of the resistant parasites. Besides the decrease in proliferation, such loss of fitness has been related to incomplete in vitro metacyclogenesis, reduced intracellular proliferation and decreased stress response, as well as in vivo decreased parasite infectivity and dissemination [31,32,51].

### 3.2. Miltefosine-Resistant Line and Wild-Type Strain Are Clearly Separated by Differences in Their Protein Abundances 

To shed light on the molecular processes involved in the emergence of resistance to miltefosine, we conducted an in-depth quantitative comparison between the proteome of the miltefosine-resistant line and the parental wild-type *L. infantum* strain. Whole cell lysates of four and three independent biological replicates, respectively, were processed by MED-FASP and analyzed by LC-MS/MS. A total of ~57,000 peptides corresponding to 5699 protein groups were identified, encompassing ~70% of the *L. infantum* predicted proteome (~8500 protein-coding genes predicted, considering one protein per gene, JPCM5 reference strain) [52,53]. Approximately 85% (4874) of protein groups were identified with at least three peptides and ~70% (3853) with at least five peptides (Figure S1, Table S1). Pearson’s correlation analysis revealed high reproducibility of proteomics data among biological replicates (coefficient ≥ 97% for WT and ≥ 85% for LiR) (Figure S1). Functional annotations of *Leishmania* proteins are still scarce, barely 25%, 32% and 43% identified protein groups have any annotation for the categories of biological process, cellular component and molecular function, respectively (Table S1). For quantitative analyses, 324 proteins identified with a single peptide, even though it was unique, were removed (Table S1).

**Figure 2:**
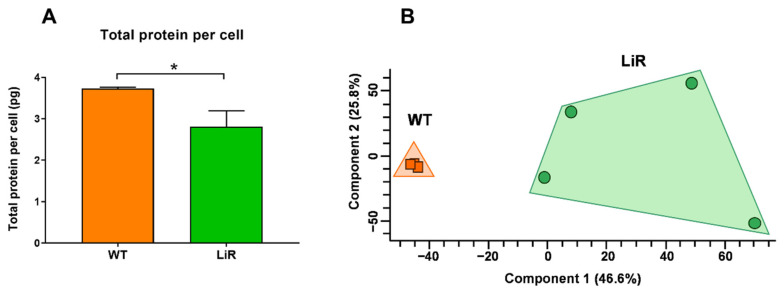
The wild-type strain is clearly separated from the miltefosine-resistant (LiR) line by differences in protein abundances. (**A**) Total protein content per cell. The total protein value per cell was determined by the histone ruler method. Bar graphs represent mean ± SD of three (WT) or four (LiR) independent experiments. Significant difference was determined by unpaired Student’s t test (* *p* < 0.05). (**B**) Principal component analysis (PCA) of protein concentration values of all validated and quantified proteins. Concentrations were determined by the total protein approach method. The clusters reveal clear separation between WT and LiR groups. WT: wild-type strain, *n* = 3; LiR: resistant line, *n* = 4.

Total protein contents, protein concentrations and protein copy numbers were calculated for 5375 proteins using the total protein approach (TPA) and the histone ruler methods as previously described [42,46]. Estimation of total protein contents were based on the genome content (32.8 Mb) reported for *L. infantum* reference strain JPCM5 [53]. First, we calculated the total DNA content in pg, which corresponds to 0.036 pg per haploid genome. From this value, we assume a diploid state for both the wild-type and the LiR line. We observed that parental *L. infantum* WT strain contains 3.74 ± 0.03 pg of protein per cell, which agrees with the total protein values previously reported for other *Leishmania* species [42,43,54] (Table S1). Remarkably, miltefosine-resistant line LiR contains 2.81 ± 0.38 pg protein representing a significant 25% decrease in the total protein per parasite (Figure 2A). A summary of general information about the proteomes of WT strain and LiR line is showed in Table S2. It has been reported that changes in the homeostatic protein content of cells may be due to changes in cell volume as well as alteration in protein synthesis [55]. In addition, it was shown that promastigotes of a miltefosine-resistant *L. infantum* line have a longer and more slender morphology under drug pressure [32]. Consistently, we observed that LiR parasites had a leaner and smaller body when compared to the wild-type strain (data not shown), so the parasite body volume could be also different. Thus, the decrease in total protein content observed in miltefosine-resistant LiR parasites could be associated with the reduction in their body cell volume. Although the total protein calculations presented here could vary according to the parasites’ ploidy state, as we are unaware of this information, we assumed a diploid state in both populations. This assumption was based on a recent publication that analyzed a large number of *L. donovani* and *L. infantum* isolates and showed that they are disomic in general, with occasional trisomy for chromosomes 23, 8 and 9 and tetraploidy, already expected, for chromosome 31 [56].

Statistical validation of protein identifications was made with Perseus software. A total of 5223 proteins were validated and these were used for subsequent statistical analyses. Principal component analysis (PCA) of protein concentrations showed a very compact cluster for the set of biological replicates of the wild-type strain and revealed a clear separation of them from those of the resistant LiR line, despite these replicates being more dispersed among each other (Figure 2B). It has been reported that drug pressure is an important source of variability for *L. donovani* [57,58], thus, the variation observed among LiR replicates could be explained by the selection process under miltefosine pressure. However, the replicates of this resistant line form a clearly separate group from the wild-type strain, confirming the existence of significant differences in protein abundance between the groups.

Consistent with previous reports [42], protein concentration values span six orders of magnitude, and tubulins, histones, elongation factor 1-alpha, heat shock proteins, kinetoplastid membrane protein-11, calmodulin and some ribosomal proteins are among the top 20 most abundant proteins (Table S1).

### 3.3. The Overall Abundance of Mitochondrial Proteins, Flagellum/Cytoskeleton Proteins and Membrane Proteins Were Increased in Miltefosine-Resistant Parasites 

**Figure 3:**
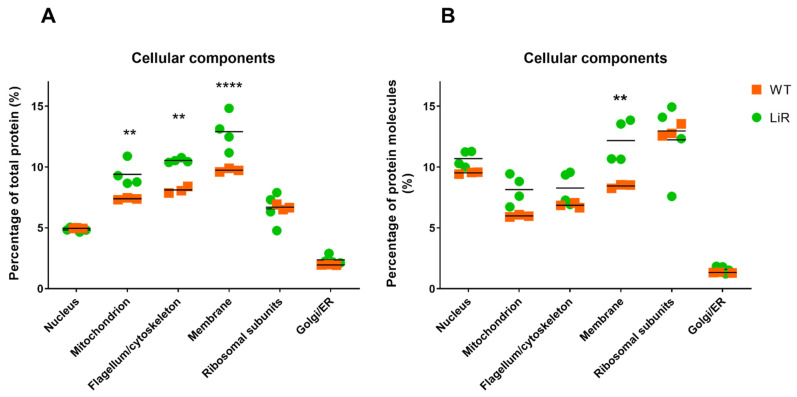
Major cellular components in terms of total protein fraction per cell and total number of copies per cell. (**A**) The percentage of total protein of each cellular component was calculated from the sum of the total protein fraction of each protein that had annotation for that component. (**B**) Percentage protein copies number (from the total protein molecules) with annotation for the cellular component. WT: wild-type strain, *n* = 3; LiR: resistant strain, *n* = 4. Significant differences between WT and LiR parasites were determined by two-way ANOVA followed by Sidak’s multiple comparisons test (** *p* < 0.01; **** *p* < 0.0001).

Using the total protein values, we calculated the copy number of each protein in both WT and LiR and estimated the total number of proteins per parasite to be: 6.01 ± 0.02 × 10^7^ molecules for WT and 4.3 ± 0.62 × 10^7^ molecules for LiR. Statistical analysis shows that this difference is significant (*p* < 0.01). This difference is consistent with the smaller size of the resistant parasites and is also reflected in the total protein fraction (%) and number of copies of the proteins that make up some of the main cellular components of the parasites (Figure 3). This analysis also allowed us to provide an overview of the abundance of the different components of the *L. infantum* architecture and to compare them between WT and LiR parasites. Based on available gene ontology annotations, nuclear proteins, ribosomal subunits and Golgi/endoplasmic reticulum proteins make up 4.9%, 6.6% and ~2.1%, respectively, of the total protein mass of the parasites in both groups (Figure 3A). In contrast, membrane (integral membrane components), flagellum/cytoskeleton and mitochondrion showed significant differences between wild-type and resistant parasites, making up 9.7%, 8.1% and 7.3% of the total protein mass, respectively, of WT, and 13%, 10.5% and 9.4%, respectively, of LiR parasites (Figure 3A). Thus, miltefosine-resistant parasites have a significantly higher percentage (in relation to total protein) of mitochondrial, flagellum/cytoskeleton and membrane proteins than WT parasites, which is also in agreement with the size reduction in LiR parasites. However, it is important to mention that due to the lack of functional annotation of a large part of the *L. infantum* proteome, this analysis may have a bias, underestimating the percentages that represent each cellular component. 

Interestingly, resistant parasites also have a higher percentage of copy number of proteins annotated as membrane components: we observed ~5 million molecules equivalent to 8.4% in the wild-type strain and 12% in the resistant line (Figure 3B), reinforcing the relevance that these organelles have for the adaptation of the parasites to the drug challenge. While still considering the number of copies, we observed that both strains showed ~5 million copies of nuclear proteins, equivalent to ~10% of the total protein molecules per parasite; 3.5 million mitochondrial proteins (~6–8% of total protein molecules), ~4 million flagellum/cytoskeleton-associated molecules (~7% of total molecules), ~5–7 million copies of ribosomal proteins (~12% of the total molecules) and ~700 thousand molecules (~1.4% of the total molecules) attributed to the Golgi apparatus/endoplasmic reticulum (Figure 3B).

### 3.4. There Are Significant Differences in Protein Abundance between the Wild-Type Strain and the Miltefosine-Resistant Line 

**Figure 4:**
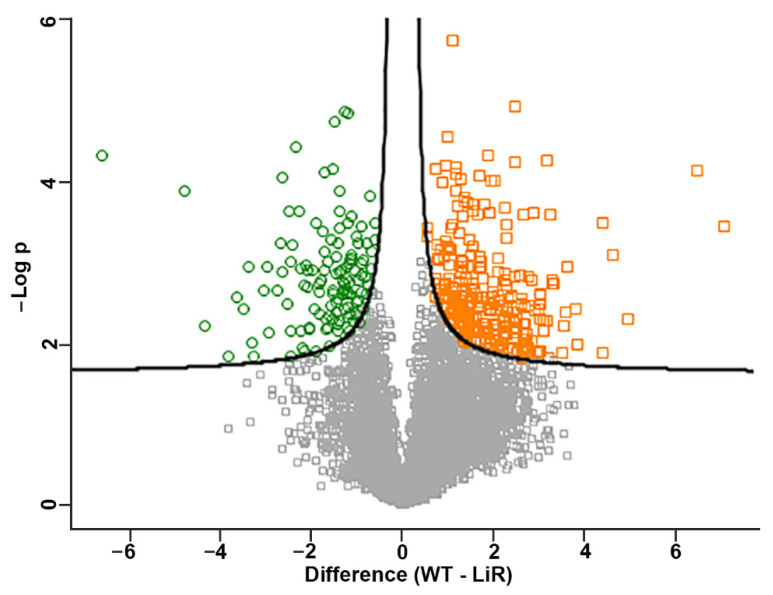
Volcano plot representation of the significant differences in protein concentration values between the wild-type *L. infantum* strain and the miltefosine-resistant line. In green, proteins with higher concentration in the resistant parasites (LiR); in orange, proteins with higher concentration in wild-type parasites (WT). The plotted values represent the average concentration of replicates from each strain. WT: wild-type strain, *n* = 3; LiR: resistant strain, *n* = 4. Significance was determined by *t*-test with 3% FDR.

The statistical significant differences in protein abundance between the WT and LiR parasites were determined by Student’s *t* test at FDR of 3%. The concentrations of 327 proteins were significantly different between the wild-type strain and the resistant line (Figure 4, Table S3). Among these proteins, 123 were more abundant in the resistant line compared to WT parasites whereas 204 proteins had lower concentrations. It is also worth noting that 81 differential proteins are uncharacterized or hypothetical and several of them showed concentration differences of more than 10-fold (Table S3). 

Further, we analyzed whether there was any group of proteins particularly enriched among those proteins with differential abundance. For this, we used the Tritrypdb enrichment tool (http://tritrypdb.org, v55, accessed on 5 February 2022) evaluating which terms of gene ontology and annotations of metabolic pathways were enriched. First, we observed significant enrichment of several categories of molecular function: *oxidoreductase activity* (2.34-fold), *proton transmembrane transporter activity* (4.04-fold) and *proton-transporting ATP synthase activity* (6.92-fold). In addition, we observed that biological processes such as *generation of precursor metabolites and energy* (3.64-fold), *proton transmembrane transport* (3.77-fold), *ATP biosynthetic process* (4.79-fold), *transmembrane ion transport* (3.63-fold), *cellular respiration* (4.61-fold), *respiratory electron transport chain* (5.93-fold) and *fatty acid derivative catabolic process* (13.84-fold), among others, are significantly overrepresented among differentially abundant proteins. Furthermore, we observed that annotations of cellular components such as *membrane protein complex* (2.52-fold), *cytochrome complex* (11.86-fold), *respirasome* (5.54-fold) as well as *respiratory chain complex* (5.54-fold) and *proton-transporting ATP synthase complex* (5.1–7.8-fold) were significantly enriched in our dataset (Table S4). Interestingly, these proteins are increased in the resistant line (Table S4). These results suggest that mitochondrial respiration, energy metabolism and membrane transport are distinct between WT and LiR parasites. These results also corroborate our findings of significant differences between WT and LiR parasites in the total protein abundance of integral membrane molecules and mitochondrial proteins shown in Figure 3. 

### 3.5. Proteins Involved in Oxidative Phosphorylation Are Significantly Increased in Resistant Parasites

**Figure 5:**
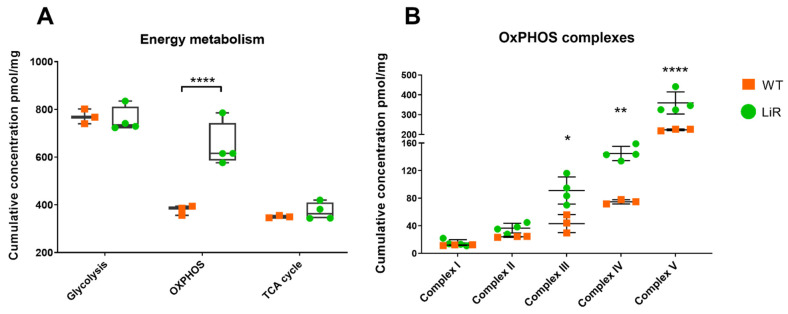
Oxidative phosphorylation metabolic pathway is significantly increased in miltefosine-resistant *L. infantum* parasites. (**A**) Sum of the abundance of proteins involved in the main pathways of energy production. (**B**) The dot plot shows the sum of the concentrations of the proteins involved in each oxidative phosphorylation complex. WT: wild-type strain, *n* = 3; LiR: resistant strain, *n* = 4. Significant differences observed between WT and LiR parasites were determined by two-way ANOVA followed by Sidak’s multiple comparisons test (* *p* < 0.05; ** *p* < 0.01; **** *p* < 0.0001). OXPHOS: oxidative phosphorylation; TCA: tricarboxylic acid. Complex I: NADH-ubiquinone oxidoreductase; complex II: succinate ubiquinone oxidoreductase; complex III: ubiquinol:cytochrome c oxidoreductase; complex IV: cytochrome *c* oxidase; complex V: FoF1-ATP synthase. Each dot represents the total sum of the concentration values of proteins involved in those processes or complexes in each biological replicate.

The results of GO enrichment led us to focus on the comparative analysis of the main pathways of energy metabolism in WT and LiR parasites. Quantitative analysis of the proteomes allowed us to determine the abundance of proteins involved in these pathways in terms of absolute concentration (Table S3). First, we analyzed the total contribution of proteins involved in glycolysis, oxidative phosphorylation and the TCA cycle (Figure 5). Miltefosine-resistant parasites show a significant increase in the absolute concentration of proteins involved in oxidative phosphorylation (OxPHOS)/mitochondrial respiration, whereas no difference was observed in the cumulative concentration of proteins involved in glycolysis or the TCA cycle between groups (Figure 5A). Then, we took a closer look at the abundance changes in the proteins that make up the respiratory complexes, to identify whether any particular complex is more or less modulated by the drug. We observed that the cumulative concentration of proteins involved in complexes III, IV and V of oxidative phosphorylation are significantly higher in the resistant LiR line than in the WT strain (Figure 5B). These results show that the proteins of *L. infantum* involved in OxPHOS are modulated in response to the selection pressure for miltefosine resistance.

**Figure 6:**
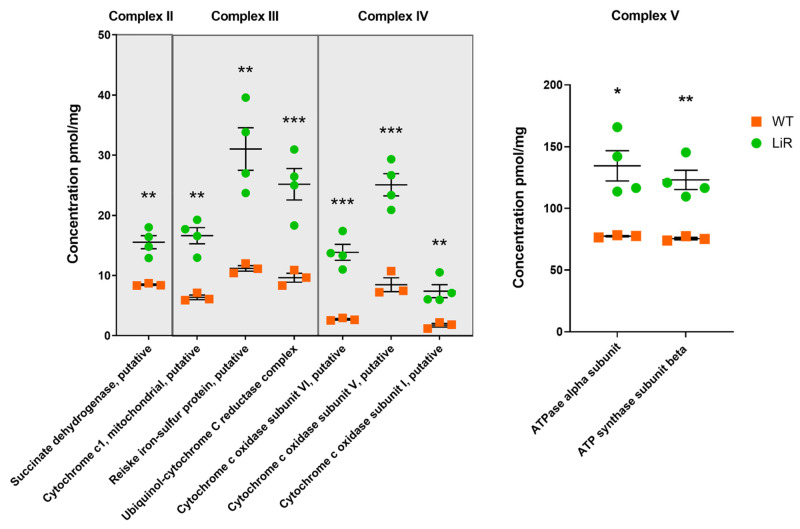
Miltefosine-resistant parasites exhibit increased concentration of enzymes involved mitochondrial respiration complexes. The dot plot shows absolute concentration values for some of the enzymes of each respiratory complex with greater abundance in resistant parasites. Significant differences observed between WT and LiR parasites were determined by *t*-test at FDR 0.03 using Perseus software and confirmed in GraphPad using the Holm–Sidak method. (* *p* < 0.05; ** *p* < 0.01; *** *p* < 0.001). WT: wild-type strain, *n* = 3; LiR: resistant strain, *n* = 4.

It is important to highlight that our proteomics approach allowed the identification and quantification of a significant number of components of the OxPHOS complexes in *L. infantum* that had not been detected in previous proteomic studies of this species, including, but not limited to, more than 11 components/subunits of complex IV and six subunits of complex V (Table S3). Proteins such as *succinate dehydrogenase* (complex II) had a 2-fold increase in LiR parasites (WT: ~8 pmol/mg—LiR: ~15.5 pmol/mg), *cytochrome c1*, *reiske iron-sulfur protein* and *ubiquinol-cytochrome_C_reductase_complex_14kD_subunit* (complex III), almost tripled their concentration in resistant parasites in relation to wild-type ones, going from ~6, ~11 and ~9 pmol/mg, respectively, in WT to ~16, 31 and ~25 pmol/mg, respectively, in LiR parasites (Figure 6, Table S3). Interestingly, we also observed that several subunits of the cytochrome *c* oxidase complex (complex IV) tripled or quintupled their concentration in the resistant parasites. For example, subunit VI went from ~3 pmol/mg in wild-type parasites to ~14 pmol/mg in the resistant ones, and subunits V and I went from ~8 pmol/mg to 25 pmol/mg and from 1.7 pmol/mg to 7.4 pmol/mg, respectively. Finally, several ATP synthase subunits (complex V) practically doubled their concentration in resistant parasites, as in the case of alpha and beta subunits that increased from ~70 pmol/mg in wild parasites to ~130 pmol/mg in resistant parasites (Figure 6, Table S3). Thus, these results suggest that miltefosine-resistant parasites have a highly active oxidative phosphorylation.

The leishmanicidal mechanism of miltefosine involves a mitochondrial dysfunction [59] together with deleterious effect on the OxPHOS pathway due mainly to the inhibition of cytochrome *c* oxidase [60]. Inhibition of this complex results in a reduction in the oxygen consumption rate as well as a significant decrease in the intracellular ATP levels of the parasites [60]. As cytochrome *c* oxidase is a main target of miltefosine, the emergence of resistance could involve, among other possibilities, the selection of parasites capable of overcoming the inhibition of this complex by increasing the abundance of its subunits, as showed here. Thus, our findings are consistent with previous reports of gene expression data showing that miltefosine-unresponsive *L. donovani* has more efficient oxidative phosphorylation [61]. It is important to note that this phenotype was selected in vitro during ~6 months of continuous passages in the presence of increasing amounts of the drug. In addition, the increase in other complexes, including ATP synthase, suggests that parasites selected for resistance to miltefosine need and/or produce/consume more ATP than wild-type parasites; however, further studies need to be conducted to prove this hypothesis. Remarkably, it is worth noting that *L. infantum* isolates that are naturally resistant to miltefosine also exhibited increased abundance of ATP synthase [24], reinforcing the idea of a very active oxidative phosphorylation pathway and ATP production. 

Additionally, based on the increased abundance of OxPHOS complexes described above, it is reasonable to suggest that the electron transport chain in resistant parasites is also very active and that the availability of reducing equivalents produced during mitochondrial catabolic pathways should also be increased in the resistant line. To have indirect evidence of this, we analyzed the abundance levels of citrate synthase and other enzymes of the TCA cycle, as well as pyruvate dehydrogenase and fatty acid β-oxidation enzymes, which are important sources of reducing molecules for fueling the electron transport chain. We also analyzed the concentration levels of glycerol-3-phosphate dehydrogenase, which could also serve as an electron entering point to the respiratory chain [62]. 

**Figure 7:**
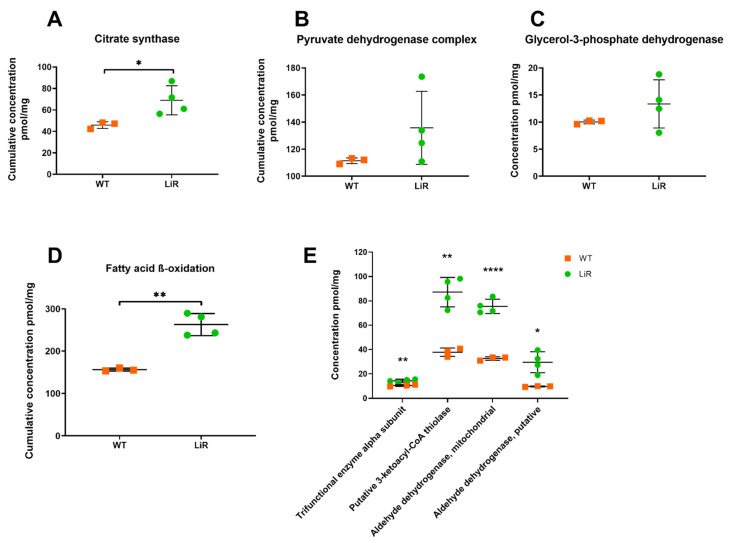
Miltefosine-resistant parasites exhibit an increased concentration of citrate synthase and fatty acid β-oxidation pathway. (**A**) Cumulative concentration of citrate synthase, significance was determined by *t*-test using the Holm–Sidak method (* *p* < 0.05); (**B**) Cumulative concentration of pyruvate dehydrogenase complex; (**C**) Absolute concentration glycerol-3-phosphate dehydrogenase; (**D**) Cumulative concentration of proteins involved in fatty acid β-oxidation pathway, significance was determined by *t*-test using the Holm–Sidak method (** *p* < 0.01); (**E**) Some of the enzymes involved in the fatty acid β-oxidation pathway with greater abundance in resistant parasites, significant differences observed between WT and LiR parasites were determined by *t*-test at FDR 0.03 using Perseus software and confirmed in GraphPad using the Holm–Sidak method (* *p* < 0.05; ** *p* < 0.01; **** *p* < 0.0001). WT: wild-type strain, *n* = 3; LiR: resistant strain, *n* = 4.

Whereas the differences in concentration levels of pyruvate dehydrogenase complex and glycerol-3-phosphate dehydrogenase were not significant between WT and LiR parasites, we observed that cumulative concentration of citrate synthase is significantly increased in LiR parasites exhibiting ~70 pmol/mg, whereas WT strain had ~46 pmol/mg (Figure 7). According to our proteomics dataset, although concentration values of isocitrate dehydrogenase and oxoglutarate dehydrogenase are lower in LiR parasites compared to WT ones, the differences were not significant (Table S3). In addition, both the cumulative concentration of the fatty acid β-oxidation pathway and the abundance of some of its key enzymes are higher in resistant parasites than wild-type ones (Figure 7). It is worth noting that the concentration of thiolase, the enzyme that catalyzes the final step of the pathway, producing acetyl-CoA and NADH, has a ~2-fold increase in resistant parasites: from 38 pmol/mg in WT to 87 pmol/mg in LiR parasites. Thus, our results suggest that miltefosine-resistant parasites maintain the integrity of the electron transport chain and higher ATP production through complex V via an increased NADH/FADH2 generation mediated by a functional TCA cycle and an enhanced activity of the fatty acid β-oxidation pathway.

**Figure 8:**
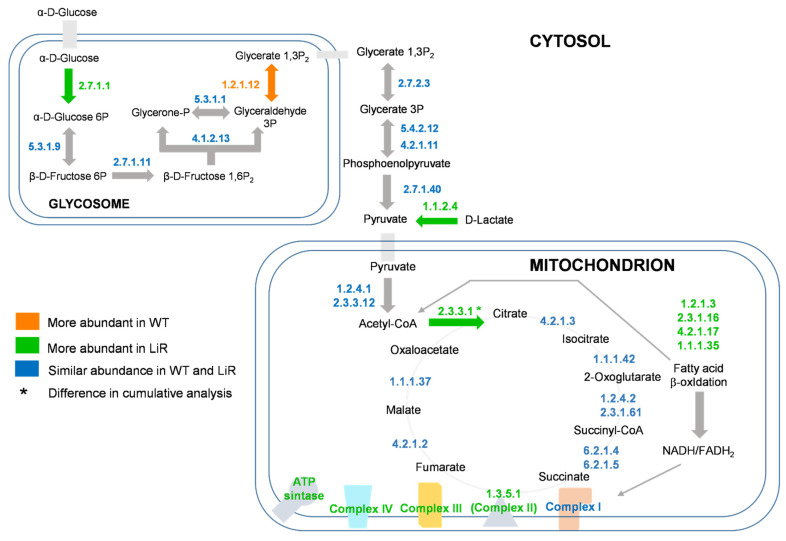
Increased oxidative phosphorylation and associated energy metabolism remodeling in miltefosine-resistant parasites. In green, enzymes that are statistically more abundant in miltefosine-resistant parasites—LiR; in orange, enzymes statistically more abundant in wild-type parasites—WT; in blue, enzymes with similar abundance in WT and LiR. Arrows are colored when the enzyme involved in that step exhibited significant change. Enzymes are represented by E.C. number. WT: wild-type strain, *n* = 3; LiR: resistant strain, *n* = 4.

Previous proteomics studies showed that miltefosine-resistant parasites exhibited decreased abundance of pyruvate dehydrogenase complex and TCA cycle, suggesting that acetyl-CoA must come from routes other than pyruvate oxidation including the fatty acid β-oxidation pathway, the breakdown of ketogenic amino acids and from acetate by an acetyl-CoA synthase [36]. Thus, based on the increased abundance described above, our results agree with this proposition, showing that the fatty acid β-oxidation pathway may be contributing with acetyl-CoA to fuel TCA. In addition, we also observed that concentration of acetyl-CoA synthase was maintained in LiR parasites at similar levels than that observed in the WT strain (Figure S2). Remarkably, we also observed a significant increase in the concentration of a D-lactate dehydrogenase (D-LDH) in miltefosine-resistant parasites (Figure S2). This enzyme converts D-lactate produced via methylglyoxal to pyruvate, which could also fuel the TCA cycle [63,64]. Interestingly, the increased abundance of D-LDH observed here agrees with recent reports showing that transcript levels of *D-Ldh* are increased in *L. donovani* parasites selected for paromomycin resistance as well as the concentration levels of this protein are increased in a *L. braziliensis* strain resistant to nitric oxide [43,65]. Thus, here we showed that in addition to the pyruvate dehydrogenase complex and β-oxidation pathway concurring for acetyl-CoA production for fueling TCA cycle, this metabolite could also be supplied by acetyl-CoA synthase and by increased production of pyruvate from D-lactate mediated by a D-LDH (Figure 8). 

### 3.6. Miltefosine-Resistant Parasites Have a Lower Concentration of Proteins Canonically Involved in Oxidative Stress Response While Exhibiting Elevated Abundance of Sterol Biosynthesis Enzymes

In drug-susceptible parasites, miltefosine can lead to an increase in the production of reactive oxygen species (ROS) resulting in cell death. In resistant parasites, this increase in ROS has not been observed, suggesting that they are more efficient in managing these species, blocking or compensating for the effects of the drug on ROS production [23,36,61,66]. Overexpression of mitochondrial iron superoxide dismutase-A (FeSODA) in *L. donovani* has been associated with protection against programmed cell death induced by the deleterious effects of miltefosine on the mitochondrion [67]. Furthermore, an *L. donovani* strain, naturally resistant to miltefosine, shows increased FeSODA [66]. This report contrasts with a recent study showing that *L. infantum* mutant clones with lower expression of FeSODA are ~2.5-fold more resistant to miltefosine [68]. In this context, we analyzed the concentration values of the proteins involved in the detoxification of ROS and cell redox homeostasis in WT and LiR parasites.

**Figure 9:**
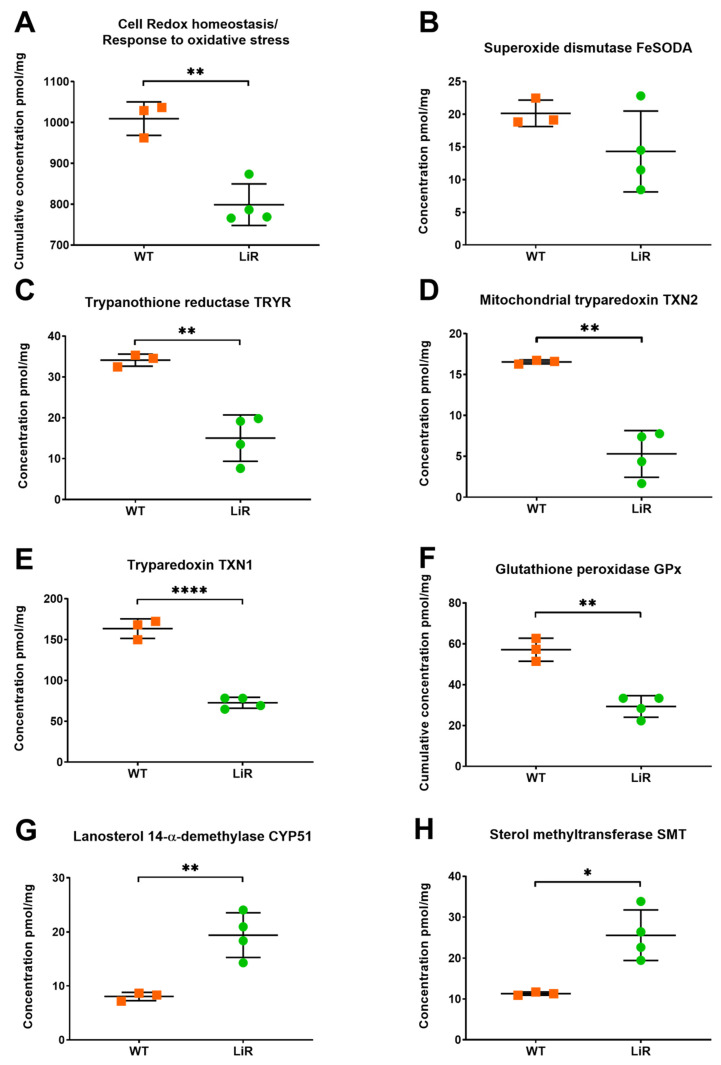
Miltefosine-resistant parasites exhibit lower cumulative concentration of proteins involved in oxidative stress response/cell redox homeostasis and increased abundance of sterol biosynthesis enzymes. (**A**) Cumulative concentration of proteins canonically involved in oxidative stress response/cell redox homeostasis; significance was determined by *t*-test using the Holm–Sidak method (** *p* < 0.01). Absolute concentration values of (**B**) mitochondrial iron superoxide dismutase-A, FeSODA; (**C**) Trypanothione reductase TRYR; (**D**) Mitochondrial tryparedoxin TXN2; (**E**) Tryparedoxin TXN1; (**F**) Glutathione peroxidase GPx; (**G**) Lanosterol 14-α-demethylase CYP51; (**H**) Sterol methyltransferase SMT. Significant differences observed between WT and LiR parasites were determined by *t*-test at FDR 0.03 using Perseus software and confirmed in GraphPad using the Holm–Sidak method (* *p* < 0.05; ** *p* < 0.01; **** *p* < 0.0001). WT: wild-type strain, *n* = 3; LiR: resistant strain, *n* = 4.

In agreement with the inverse relation between FeSODA expression and miltefosine resistance, our in-depth proteomics dataset revealed no significant difference in FeSODA abundance and even a decrease in its concentration in LiR parasites (Figure 9). In addition, other proteins involved in the oxidative stress response/cell redox homeostasis were more abundant in WT parasites than in miltefosine-resistant ones (Figure 9). In fact, we observed that wild-type parasites have a higher cumulative concentration of proteins involved in these processes and a significantly higher individual concentration of trypanothione reductase (TRYR), glutathione peroxidase (GPx), tryparedoxin (TXN1) and mitochondrial tryparedoxin (TXN2) (Figure 9), among others. These results suggest that miltefosine-resistant *L. infantum* parasites must have other mechanisms to deal with the production of ROS. 

Recently, it was reported that *L. major* null mutants for sterol 14-α-demethylase (CYP51) or sterol methyltransferase (SMT), enzymes of the biosynthesis of sterols, present altered mitochondrial membrane potential, impaired respiration and significant mitochondrial ROS accumulation [69,70]. In addition, it has been demonstrated that CYP51 is indispensable in *L. donovani* growth [71]. Remarkably, we observed significantly higher abundance of CYP51 and SMT in miltefosine-resistant parasites (Figure 9). Concentration of CYP51 increased from 8 pmol/mg in WT parasites to 19.4 pmol/mg in LiR ones, whereas the protein concentration of SMT augmented from 11.3 pmol/mg in WT to 25.6 pmol/mg in LiR (Figure 9). Then, it is plausible to suggest that CYP51 and SMT could play important roles in ROS detoxification in the miltefosine-resistant *L. infantum* line. However, further studies should be conducted to demonstrate this hypothesis. 

### 3.7. Concentration Levels of ABC Transporters and a Phospholipid Transporting ATPase Involved in Miltefosine Resistance Are Significantly Different between WT and LiR Parasites

**Figure 10:**
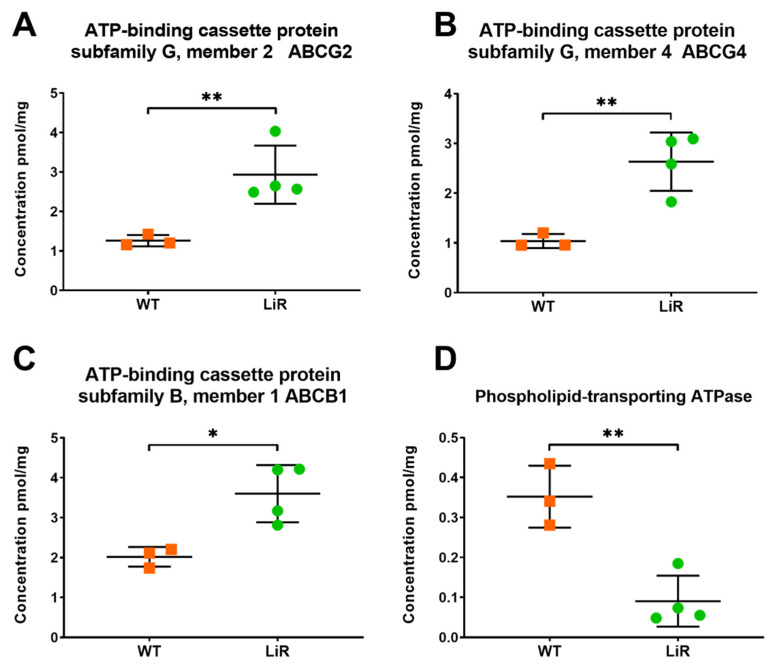
Membrane transporters involved in the reduction in drug accumulation into the parasites are significantly different between wild-type and miltefosine-resistant parasites. Absolute concentration of ABC transporters involved in drug efflux: (**A**) ABCG2; (**B**) ABCG4; (**C**) ABCB1, or in lower drug influx; (**D**) Phospholipid-transporting ATPase. Significant differences observed between WT and LiR parasites were determined by *t*-test at FDR 0.03 using Perseus software and confirmed in GraphPad using the Holm–Sidak method (* *p* < 0.05; ** *p* < 0.01). WT: wild-type strain, *n* = 3; LiR: resistant strain, *n* = 4.

The ATP-binding cassette (ABC) transporters comprise a superfamily of transmembrane proteins that are involved in the transport of different molecules, from ions to large polypeptides, which have been largely recognized by their activity as drug efflux pumps [72]. In *Leishmania* spp., ABC transporters present high diversity and several of them have been mainly implicated in drug resistance [73]. Indeed, overexpression of ABC transporters is involved with miltefosine extrusion in resistant parasites [74,75]. Although quantification of ABC transporters has being challenging because they are low-abundant transmembrane proteins, the sample processing and quantitative methods used here allowed us to identify and quantify 34 (out of 42) ABC transporters in our *L. infantum* samples (Table S3). We observed that transporters ABCB1, ABCG2 and ABCG4 have a significantly higher concentration in resistant parasites, being 2- to 2.5-fold more abundant compared to the wild-type (Figure 10). The ABCB1 transporter from *L. tropica* conferred resistance to alkyl phospholipids and reduced the accumulation of a fluorescent lipid analogue of phosphatidylcholine [74,76]. In *L. major*, the ABCG2 transporter plays a role in the transport of non-protein thiols and phosphatidylserine, as well as in parasite virulence, resistance to antimonials and redox metabolism [77,78]. In turn, the ABCG4 transporter of *L. infantum* has been reported to participate in the active outward transport of phosphatidylcholine, alkyl-glycerophosphocholine and phosphocholine derivatives, therefore, contributing to the miltefosine resistance phenotype [75]. The increased abundance of those transporters would reduce the intracellular accumulation of miltefosine due to active drug efflux in LiR parasites.

In addition, it has been well described that drug-resistant parasites have mutations in the gene encoding a P-type ATPase of the phospholipid translocase subfamily, which result in decreased expression of this ATPase and, consequently, in a lower influx of miltefosine in resistant parasites [79]. In line with this information, we observed a significant decrease in that phospholipid transporting ATPase in resistant parasites (Figure 10), which could result in a diminished miltefosine influx in these parasites. This finding is interesting, as it would indicate that drug pressure leads to a decrease in the concentration of this translocase; however, we cannot say that a decrease was due to mutations in the coding sequence and further experiments would be necessary to answer that question. Together, these findings show that miltefosine-resistant parasites can modulate the abundance of different membrane transporters in order to reduce drug accumulation into the cell interior through active efflux and lower influx of the drug.

## 4. Conclusions

Our study provides for the first time large-scale quantitative proteomics data on miltefosine resistance in *L. infantum* promastigotes. More than 5600 protein groups were identified and quantified, allowing for an unbiased and deeper analysis of the molecular changes that contribute to the resistance phenotype. We describe the changes in protein abundance between the wild-type strain and the resistant line derived by in vitro selection. These differences were described both in number of protein copies per cell and in absolute concentration of proteins. We observed that resistance is selected through a complex adaptation response of the parasites that involves a remodeling of integral components of the membrane, flagellum/cytoskeleton and mitochondria, as well as a significant increase in oxidative phosphorylation complexes, with particular emphasis on complex IV and ATP synthase accompanied by a potential increase in energy metabolism mainly dependent on the β-oxidation of fatty acids. We observed that proteins canonically involved in ROS detoxification were not modulated or were even decreased in resistant parasites, indicating that other pathways participate in this process. We also detected a potential contribution of sterol biosynthesis enzymes to this resistance phenotype and changes in the abundance of proteins known to be involved in miltefosine resistance such as ABC transporters and phospholipid transport ATPase. Together, our data show a more complete picture of the elements that make up the phenotype of in vitro selected resistance to miltefosine in *L. infantum*.

## Data Availability

The mass spectrometry proteomics data have been deposited to the ProteomeXchange Consortium via the PRIDE [48] partner repository with the dataset identifier PXD031912.

## Supplementary Materials

The following supporting information can be downloaded at: https://www.mdpi.com/article/10.3390/proteomes10020010/s1, Figure S1: Peptide coverage and protein abundance correlation among replicates. (A) 85% proteins were identified with at least three peptides. Multiscatter plots with Pearson’s correlation coefficient of protein abundance among (B) Wild-type (WT) *L. infantum* strain replicates; (C) miltefosine-resistant (LiR) *L. infantum* line replicates. Plotted values represent the total protein fraction of each individual protein calculated by the total protein approach; Figure S2: Abundance of acetyl-CoA synthase and D-lactate dehydrogenase in WT and LiR. (A) Absolute cumulative concentration of acetyl-CoA synthase; (B) absolute concentration values of D-lactate dehydrogenase. Significant differences observed between WT and LiR parasites were determined by t-test at FDR 0.03 using Perseus software and confirmed in GraphPad using the Holm–Sidak method (* *p* < 0.05). WT: wild-type strain, *n* = 3; LiR: resistant strain, *n* = 4; Table S1: Protein Groups identified; Table S2: Summary of general information about the proteomes of *Leishmania infantum* wild-type strain and miltefosine-resistant line; Table S3: Statistical differences between groups; Table S4: Gene ontology enrichment.
