# Supplementary material for: In-Depth Quantitative Proteomics Characterization of In Vitro Selected Miltefosine Resistance in Leishmania infantum
Source: Proteomes. 2022 Mar 31;10(2):10. doi: 10.3390/proteomes10020010 (PMC9036279; doi:10.3390/proteomes10020010)
Supplement: Supplementary file 1 [file proteomes-10-00010-s001.zip › Table S2.pdf]

Table S2. Summary of general information about the proteomes of miltefosine-resistant *Leishmania infantum* line selected in vitro and the parental *L. infantum* wild-type strain.

	**LiR**<br>**Miltefosine-resistant line**	**WT**<br>**Parental strain**
**Biological replicates**	4	3
**Peptides identified**	28156 ± 4512	30913 ± 664
**Protein groups identified**	5071	5435
**Total protein per cell**	2.81 ± 0.38 pg	3.74 ± 0.03 pg
**Total protein molecules per parasite**	$4.3 \pm 0.62 \times 10^7$	$6.01 \pm 0.02 \times 10^7$
