# Supplementary figures and images for: In-Depth Quantitative Proteomics Characterization of In Vitro Selected Miltefosine Resistance in Leishmania infantum
Source: Proteomes. 2022 Mar 31;10(2):10. doi: 10.3390/proteomes10020010 (PMC9036279; doi:10.3390/proteomes10020010)

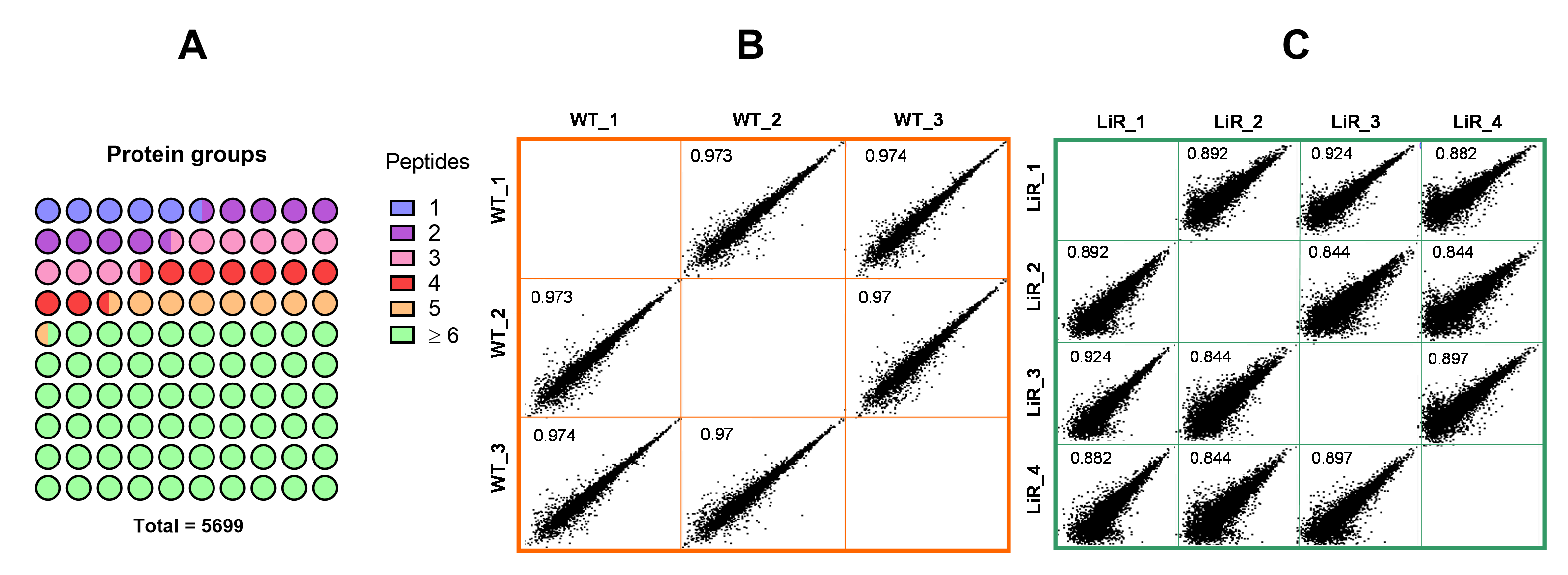

Supplement: Supplementary file 1 [file proteomes-10-00010-s001.zip › Figure S1.tif]

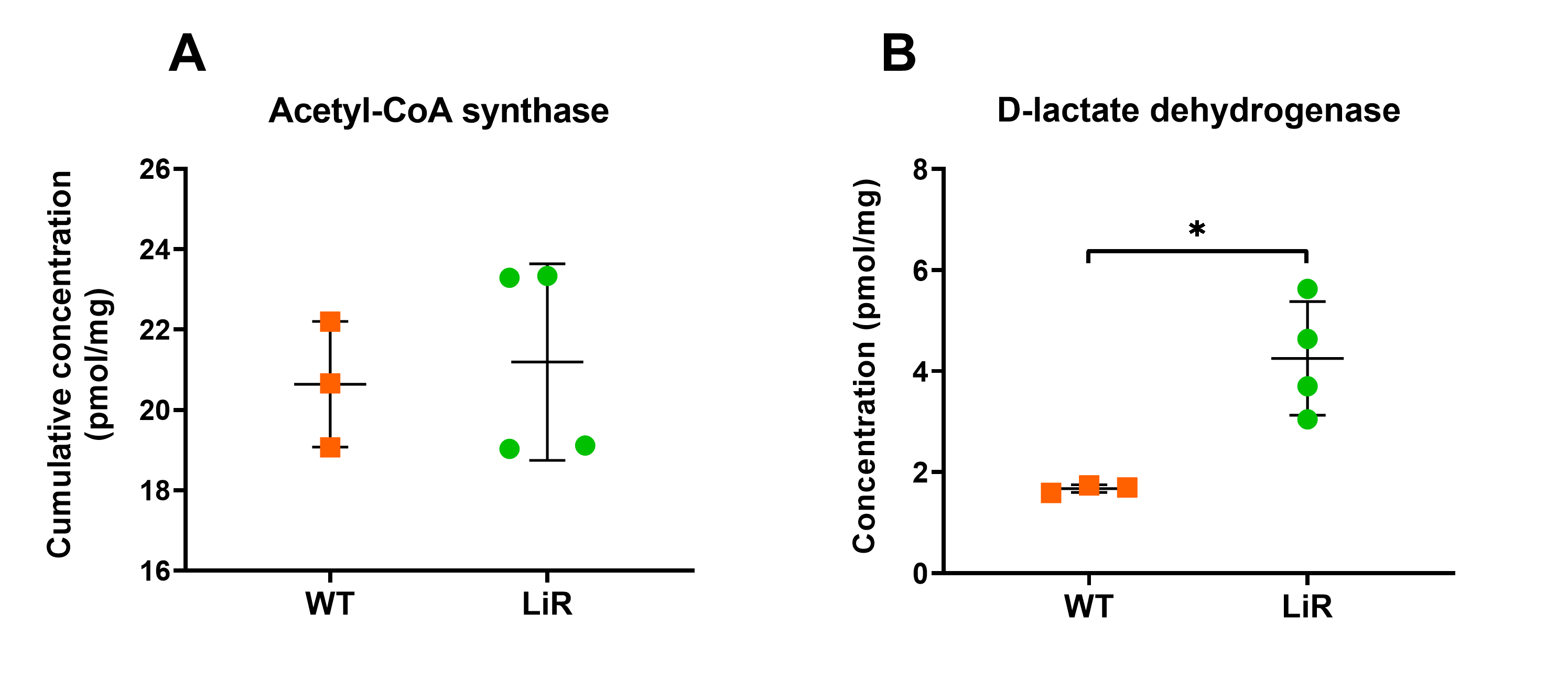

Supplement: Supplementary file 1 [file proteomes-10-00010-s001.zip › Figure S2.tif]
